# DOT1L: a new therapeutic target for aggressive breast cancer

**DOI:** 10.18632/oncotarget.5860

**Published:** 2015-09-28

**Authors:** Jeong-Yeon Lee, Gu Kong

**Affiliations:** Department of Pathology, College of Medicine, Hanyang University, Seoul, Republic of Korea

**Keywords:** Chromosome Section, DOT1L, histone methylation, EMT, breast cancer

Disruptor of telomeric silencing-1 like (DOT1L) is a histone-modifying enzyme that specifically catalyzes the mono-, di-, and tri-methylation of histone H3 on lysine-79 (H3K79). DOTlL-mediated H3K79 methylation is associated with active transcription, and it has been implicated in many biological processes including DNA damage response, cell cycle, and embryonic cell development [1]. Furthermore, accumulating studies have shown the emerging role of DOT1L and H3K79 methylation in the initiation and maintenance of mixed lineage leukemia (MLL)-rearranged leukemia. During leukemogenesis, MLL fusion proteins such as AF9, AF10, and ENL recruit DOT1L to their target gene loci to elevate H3K79 methylation levels, thus increasing the aberrant expression of genes that contribute to leukemic transformation of hematopoietic progenitors [2]. Thus, DOT1L has been suggested to be a promising molecular target for MLL-rearranged leukemia. Indeed, selective DOT1L inhibitors exhibit remarkable anti-tumor effects in these cells, and EPZ-5676, the most advanced DOT1L inhibitor, is currently in clinical trials for adult and pediatric patients with MLL-rearranged leukemia [2]. While the oncogenic potential of DOT1L in leukemia has been well described, there is little evidence for the role of DOT1L in solid tumors.

Recently, some studies have shown the distinct role of DOT1L in several solid tumors that do not harbor MLL translocation. In prostate cancer, DOT1L directly methylates androgen receptor to regulate its activity [3]. DOT1L also has been identified as a coactivator of Wnt signaling in colorectal cancer, but the therapeutic potential of DOT1L inhibition remains questionable due to the contradiction of DOT1L effect on Wnt-dependent gene expression [4]. A recent study shows that selective DOT1L inhibitors suppress proliferation and migration of breast cancer cells [5]. In our recent work published in *Nature Communications* [6], the advanced insights into the functional role and clinical relevance of DOT1L in breast cancer have been highlighted. DOT1L induces neoplastic transformation of immortalized human breast epithelial cells, as well as increases tumor initiation and growth of human breast cancer cells. Furthermore, DOT1L depletion inhibits the breast cancer metastasis to lung in a xenograft mouse model. Consistently, DOT1L is associated with worse outcome in aggressive human breast cancer [6]. This evidence suggests that DOT1L could function solely as an oncogene in an independent manner of MLL fusion partners in breast cancer, suggesting DOT1L to be a potential drug target for aggressive breast cancer.

Notably, these oncogenic effects of DOT1L were due to an increase of epithelial-mesenchymal transition (EMT)-induced cancer stem cell (CSC) properties via DOT1L-dependent transcriptional activation of EMT-promoting genes, such as Snail, ZEB1, and ZEB2, in human breast cancer [6]. EMT, which leads loss of cell adhesion and acquisition of cell motility, is an essential process for tumor invasion and metastasis. Moreover, EMT has been recently defined as one of major characteristics of stem-like cells in normal and malignant breast epithelial cells. Several EMT-promoting factors that inhibit epithelial marker E-cadherin transcription can induce the stemness of breast cancer [7]. The aberrant expression of these EMT regulators has been involved in promoting malignant transformation of breast epithelial cells and tumor recurrence and metastasis, suggesting them as therapeutic targets for aggressive breast cancer. Interestingly, DOT1L controls both EMT and CSCs by activating the E-cadherin repressors, Snail, ZEB1, and ZEB2 [6]. Furthermore, the enzymatic activity of DOT1L towards H3K79 methylation is critical for gene expression of these EMT modulators. Thus, DOT1L may facilitate the aggressiveness of breast cancer as a regulator of EMT-promoting factors and selective DOT1L inhibitors could be effective for inhibiting EMT and CSCs.

In the regulation of EMT-promoting genes by DOT1L, we provide mechanistic insights into novel transcriptional and epigenetic modulating functions of DOT1L in cooperation with c-Myc transcription factor. First, c-Myc is required for the recognition of DOT1L to target genes. Whether DOT1L has its own DNA-binding ability has been unclear, and in leukemia, DOT1L is mainly recruited to target gene loci by MLL-fusion proteins [2]. In breast cancers that do not have MLL translocation, c-Myc seems to function as a guide for DOT1L recognition to chromatin, since the depletion of c-Myc inhibits DOT1L recruitment to the proximal promoters of EMT genes that possess E-box motifs in breast cancer cells [6]. Second, DOT1L facilitates the formation of a c-Myc-containing transcriptional active complex. C-Myc can function as a transcriptional activator or repressor depending on its binding partners. Interestingly, When DOT1L binds to c-Myc, c-Myc preferentially interacts with p300 acetyltransferase rather than DNMT or HDAC1 transcriptional repressive components [6]. Although this biochemical mechanism should be further investigated, this evidence suggests that DOT1L is required for c-Myc-dependent transcriptional activation. Moreover, consistent with the association of DOT1L and histone acetylation in MLL-rearranged leukemia [2], histone acetylation seems to be an essential for DOTlL-depedent transcriptional activation in breast cancer.

In summary, DOT1L plays an important role in the initiation and progression of breast cancer by targeting the gene expression of EMT-promoting factors via cooperating with c-Myc/p300 transcriptional active complex in a different mechanism from leukemia, suggesting DOT1L to be a therapeutic target for aggressive breast cancer.
